# Measuring the accuracy of self-reported height and weight in a community-based sample of young people

**DOI:** 10.1186/1471-2288-12-175

**Published:** 2012-11-21

**Authors:** Anna L Bowring, Anna Peeters, Rosanne Freak-Poli, Megan SC Lim, Maelenn Gouillou, Margaret Hellard

**Affiliations:** 1 Centre for Population Health, Burnet Institute, 85 Commercial Rd, Melbourne, VIC, 3004, Australia; 2 Baker IDI Heart and Diabetes Institute, 99 Commercial Road, Melbourne, VIC, 3004, Australia; 3 Department of Epidemiology & Preventive Medicine, Faculty of Medicine, Nursing & Health Sciences, School of Public Health and Preventive Medicine, Monash University, The Alfred Centre, 99 Commercial Road, Melbourne, VIC, 3004, Australia; 4 Nossal Institute for Global Health, The University of Melbourne, Level 4, Alan Gilbert Building, 161 Barry Street, Carlton, Melbourne, VIC, 3010, Australia

**Keywords:** Body height, Body weight, Body mass index, Overweight, Obesity self-report, Validity, Young people

## Abstract

**Background:**

Self-reported anthropometric data are commonly used to estimate prevalence of obesity in population and community-based studies. We aim to: 1) Determine whether survey participants are able and willing to self-report height and weight; 2) Assess the accuracy of self-reported compared to measured anthropometric data in a community-based sample of young people.

**Methods:**

Participants (16–29 years) of a behaviour survey, recruited at a Melbourne music festival (January 2011), were asked to self-report height and weight; researchers independently weighed and measured a sub-sample. Body Mass Index was calculated and overweight/obesity classified as ≥25kg/m^2^. Differences between measured and self-reported values were assessed using paired t-test/Wilcoxon signed ranks test. Accurate report of height and weight were defined as <2cm and <2kg difference between self-report and measured values, respectively. Agreement between classification of overweight/obesity by self-report and measured values was assessed using McNemar’s test.

**Results:**

Of 1405 survey participants, 82% of males and 72% of females self-reported their height and weight. Among 67 participants who were also independently measured, self-reported height and weight were significantly less than measured height (p=0.01) and weight (p<0.01) among females, but no differences were detected among males. Overall, 52% accurately self-reported height, 30% under-reported, and 18% over-reported; 34% accurately self-reported weight, 52% under-reported and 13% over-reported. More females (70%) than males (35%) under-reported weight (p=0.01). Prevalence of overweight/obesity was 33% based on self-report data and 39% based on measured data (p=0.16).

**Conclusions:**

Self-reported measurements may underestimate weight but accurately identified overweight/obesity in the majority of this sample of young people.

## Background

In Australia, obesity is a national health priority, and approximately one quarter of young adult Australians are overweight or obese [1]. This is a concern for immediate social and health problems, as well as risk of future obesity in adulthood and associated chronic health problems, such as type II diabetes, cardiovascular diseases, musculoskeletal disease and some cancers [1]. Data on prevalence and trends in obesity in young people are needed to inform, monitor, and evaluate appropriate policy and interventions.

Body mass index (BMI) is widely used as a measure of obesity due to its simple derivation from height and weight. Height and weight are commonly self-reported in population health surveys for ease of collection [2]. In contrast to direct measurement, self-report enables a large number of individuals to be sampled at relatively minimal cost, time, and resources and the survey tool can be administered face to face, by telephone or online.

Self-reported height and weight may be affected by response or recall bias. It is commonly found that weight is underestimated while height is often overestimated [3-8], leading to underestimation of BMI [5,7,8]. Some studies have identified a greater discrepancy in self-reported and measured height and weight among certain groups, including overweight or obese individuals compared to healthy or underweight individuals [6,7,9-12], females [7-9,12,13], dieters compared to non-dieters, non-smokers compared to smokers [11,14], and older individuals [7,9,11]. Such systematic error may lead to lower prevalence estimates of overweight and obesity and systematic bias in studies examining the relationship between obesity and health outcomes dependent on self-reported measurements. In the Australian context, from a telephone-recruited population survey in Adelaide, classification of overweight/obesity among 18–24 year olds was 6.3% through self-report data and 10.1% through measured data,[13]; while in the 1995 Australian National Health Survey, classification of overweight/obesity among 15–19 year olds was 18% and 12% of males and females, respectively, through self-report data and 25% and 19% of males and females, respectively, through measured data [6].

In spite of these findings, there remains a lack of data reporting the willingness and accuracy of young people to self-report height, weight, and derived BMI, particularly in community-based settings. Traditional methods of recruiting young people into population surveys, such as random digit dialling and school-based recruitment, are subject to increasing barriers affecting participation, tracking, and retention, such as high mobility, changes in telephone use and technology, and non-school attendance [15,16]. Community-based recruitment provides an alternative means to recruit large numbers of young people, and we have previously shown how periodic recruitment from a music festival can be used for surveillance in young people [17,18]. In this setting, use of self-reported measurements would be preferable, given the substantial added time and resources required to independently measure participants.

Here we use participants in a risk behaviour survey to determine the accuracy of self-reported height and weight in Australian 16–29 year olds in a community-based setting. Specifically, the aims of this study were:

1) To determine whether young people participating in a risk behaviour survey are able and willing to provide self-reported height and weight data; and

2) To assess the accuracy of self-reported height, weight and BMI compared to measured values in a community-based sample of young people.

## Methods

### Participants and procedure

**Figure 1:**
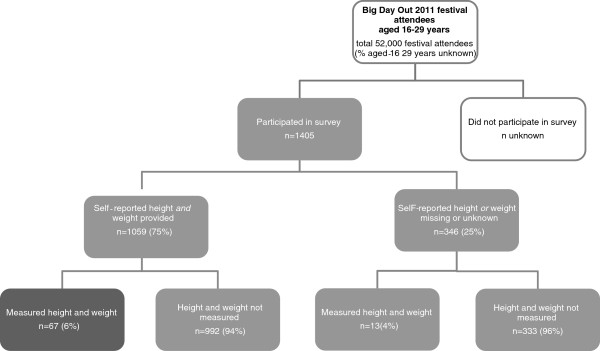
**Representation of participant recruitment for survey and independent measurement.** Final sample depicted in dark grey.

Participants were recruited at the Melbourne Big Day Out (BDO) - one day music festival in January 2011 - as part of an ongoing behavioural surveillance system that has been undertaken at the BDO since 2005 [17-19]. A convenience sample of young people aged 16–29 years was recruited by approximately 20 trained researchers (Figure 1). Participants approached or were approached by researchers in and around a study market stall in the food and market area of the festival . Recruitment occurred between 10am and 3pm. Once the survey was explained, participants self-completed a consent form and short questionnaire taking approximately 10 minutes.

The structure of the market stall set up meant there was one measuring station for height and weight. After completing the questionnaire, participants were invited to have their height and weight measured if approximately three or less participants were waiting to use the measuring station.

### Questionnaire

A core set of questions about drug and alcohol use and sexual risk behaviours is asked annually in the BDO survey [17,18]. In 2011, participants were additionally asked to self-report their height in centimetres (without shoes) and weight in kilograms (without clothes and shoes) with an option for “don’t know” in both questions.

### Measures

#### Anthropometry

When submitting their completed survey, a subset of individuals was invited to have their height and weight measured by a trained research assistant (Figure 1). As outlined above, invitation was based upon the availability of the measurement station. Participants were measured without shoes and wearing light clothes using digital scales and a portable stadiometer. Height and weight were measured to the nearest 1cm and 0.1kg, respectively.

To link measurements to the participants’ surveys, measurements were labelled using either the survey identification number or a “porn star name”, a novel anonymous identifier derived from name of first pet and name of first street [20], which was also reported in the questionnaire.

BMI for both self-reported and measured values was calculated as weight (kg) divided by height squared (m^2^). For participants aged 18 years and older, overweight and obesity were classified using the standard international adult BMI ranges: *underweight* (BMI <18.5), *healthy weight* (BMI = 18.5-24.9), *overweight* (BMI = 25.0-29.9), or *obese* (BMI >30). [21,22] For participants aged under 18 years, age and sex specific BMI cut-offs for overweight and obesity were used, as defined by the International Obesity Task Force.[23] For the purpose of this research, obesity classification was dichotomised as non-overweight (BMI<25.0) and overweight/obese (BMI≥25.0).

#### Socio-demographics indicators

A range of self-reported demographic indicators were assessed: sex (male, female); age (16–19, 20–29 years); born in Australia (yes, no); area of residence (major, non-major city); living arrangements (if live with parents/partner; yes, no); post-high school education (if completed or in the process of completing; yes, no); recreational income (<$120, ≥$120 per week). Area of residence was classified from Australian postcodes of residence using the Australian Standard Geographical Classification (ASGC) Remoteness Areas system [24]. Recreational income was defined as money available for spending or saving, not including essential living costs or tax.

#### Analysis

Data were entered into a Microsoft Access database and statistical analysis was conducted in Stata version 11 [25].

To assess factors associated with survey participants’ (n=1405) ability and willingness to provide self-report height and weight measurements, participants who did and did not self-report height and weight were compared by demographic indicators using chi-square tests of proportion (Figure 1).

Among those with self-reported height and weight (n=1059), participants with and without measurements taken were compared using chi-square tests of proportion and Wilcoxon rank-sum test.

The final analysis was limited to participants with both self-reported and measured height and weight (n=67) (Figure 1). Accuracy of self-report height, weight, and derived BMI compared to corresponding measured values was assessed in four ways:

i. Test of equality between measured and self-reported mean or median was assessed using a paired t-test or Wilcoxon signed ranks test, respectively, both overall and stratified by sex, age group, and obesity classification categories.

ii. Correlation of self-report to measured values was calculated using Pearson correlation coefficient or Spearman rank correlation coefficient according to distribution of both self-reported and measured height, weight and BMI.

iii. The proportion of individuals who accurately, under- and over- reported their weight/height was compared using a Fisher’s exact test. Accurate report of weight/height was defined as less than 2kg/2cm absolute difference between self-report and measured weight/height given an acceptable margin of error, based on previous studies [26]. Under-report of height/weight was defined as self-report ≥2cm/2kg less than measured values; over-report of weight/height was defined as self-report being ≥2cm/2kg more than measured values. The proportion of participants misreporting their height or weight by five or more kilograms was also calculated.

iv. Prevalence of overweight/obesity based on self-report and measured data was compared using two-sided exact McNemar’s test. Misclassification of overweight/obesity was measured in terms of sensitivity and specificity with exact binomial 95% confidence intervals (CI), whereby 100% indicates maximum agreement between self-reported and measured classification.

Significance level was 0.05 in all analyses.

#### Ethics

Ethical approval was granted by the Alfred Hospital Human Research Ethics Committee (number 326/08).

## Results

In total, 1405 questionnaires were completed (see Figure 1). Self-reported height was unknown in 237 (18%) and missing in 28 (2%) questionnaires; self-reported weight was unknown in 148 (11%) and missing in 13 (1%) questionnaires. Overall, 1059 (75%) participants self-reported both height and weight. Comparatively, females, 16–19 year olds, those without post-high school education, those who live with their parents, and those who did not live with a partner were significantly more likely than their counterparts to have self-reported height and/or weight unknown or missing (all p<0.01)( Table 1).

**Table 1:** Comparison of participant characteristics and risk behaviours by self-report height and weight status

	**Self-reported height and weight**		**Self-reported height and/or weight missing/unknown**		**p-value**^**1**^
	**n**	**%**	**n**	**%**	
**TOTAL**	1059	75	346	25	
**Sex**					**<0.01**
Male	436	82	99	19	
Female	623	72	247	28	
**Median age (IQR)**	19.7	(18.0-22.9)	18.3	(17.1-20.7)	**<0.01**
16-19	558	70	240	30	
20-29	501	83	106	17	
**Post high-school education**^**2**^					**<0.01**
Yes	485	80	122	20	
No	561	72	219	28	
**Lives with parent/s**					**<0.01**
Yes	669	72	266	28	
No	369	83	77	17	
**Lives with partner**					**<0.01**
Yes	125	87	19	13	
No	912	74	324	26	
**Recreational income**^**3**^					0.10
<$120 per week	703	74	245	26	
≥$120 per week	331	78	92	22	
**Area of residence**^**4**^					
Major city	697	75	232	25	0.88
Non-major city	316	75	103	25	

^1^ Chi-square test of proportions, comparing participants with self-reported height *and* weight to participants with self-reported height *or* weight *missing/unknown*.

^2^*Post-high school education* defined as having completed or in the process of completing post-high school education.

^3^*Recreational income* defined as money available for spending or saving, not including essential living costs or tax.

^4^*Area of residence classified* using Australian Standard Geographical Classification (ASGC).

Remoteness Areas (RA) system, whereby RA 1 is coded major city; RA 2–5 is coded non-major city.

Significant p-values (p<0.05) shown in boldface.

IQR - inter-quartile range

Eighty survey participants had height and weight independently measured. Amongst survey participants providing self-reported height and weight, participants with (6%) and without (94%) independent anthropometric measurement did not differ by demographic characteristics (all p≥0.08).

The final sample is based on 67 participants with complete self-reported and measured height and weight (Figure 1); 51% were male, the median age was 20.1 years, 37% had post-high school education, 67% lived in a major-city, 57% lived with their parent(s), and 58% had $120 or less recreational income per week.

### Accuracy of self-reported height, weight, and BMI compared to measured values

As a continuous variable, self-reported and measured height did not significantly differ overall (p=0.06), but mean self-reported height was 2.3 cm less than mean measured height among females (p=0.01) (Table 2). Median self-reported weight was two kilos less than median measured weight (p<0.01). When stratified by age group, sex, and obesity classification, self-reported weight remained significantly lower than measured weight in all sub-categories with the exception of males. Self-reported BMI did not differ from measured BMI overall or when stratified.

**Table 2:** Comparison of measured and self-reported values for height, weight, and BMI overall and by sex, age group, and obesity classification

			**Measured**		**Self-reported**		**Pearson correlation between measured and self-reported**	**Test of equality between measured and self-reported, p-value**
			**Mean (SD)**	**Median**	**Mean (SD)**	**Median**		
**Total**	(n=67 )	Height (cm)	173.7 (9.8)	175.0	172.7 (11.9)	174.0	0.94	0.06
		Weight (kg)	72.0 (14.7)	70.2	70.1 (14.5)	68.0	0.96*	<0.01*
		BMI (kg/m^2^)	23.7 (3.2)	23.2	23.4 (3.2)	23.0	0.84	0.16
**Sex**	Males (n=34)	Height (cm)	180.2 (7.5)	180.0	180.5 (8.5)	180.0	0.94	0.66
		Weight (kg)	80.2 (14.0)	78.8	79.2 (13.2)	80.0	0.92*	0.28*
		BMI (kg/m^2^)	24.6 (3.3)	24.5	24.2 (2.8)	23.3	0.85	0.21
	Females (n=33)	Height (cm)	166.9 (6.9)	165.0	164.6 (9.3)	164.0	0.84	**0.01**
		Weight (kg)	63.6 (9.9)	63.1	60.8 (8.8)	60.0	0.93	**<0.01**
		BMI (kg/m^2^)	22.8 (2.9)	22.0	22.5 (3.4)	21.1	0.82	0.44
**Age group (years)**	16-19 (n=33 )	Height (cm)	173.6 (11.0)	172.0	172.4 (13.6)	172.0	0.92	0.22
		Weight (kg)	70.2 (14.7)	68.0	68.1 (14.6)	65.0	0.94*	0.01*
		BMI (kg/m^2^)	23.1 (2.9)	22.0	22.8 (3.1)	22.6	0.76	0.39
	20-29 (n=34 )	Height (cm)	173.8 (8.7)	175.8	172.9 (10.2)	175.5	0.96	0.10
		Weight (kg)	73.8 (14.6)	73.3	72.1 (14.4)	70.5	0.96	**0.01**
		BMI (kg/m^2^)	24.3 (3.4)	24.7	23.9 (3.3)	24.3	0.89	0.23
**Obesity classification**	Non-overweight^1^ (n=43)	Height (cm)	172.4 (9.2)	172.0	171.3 (10.9)	172.0	0.92	0.22
		Weight (kg)	65.1 (9.6)	66.0	64.0 (10.4)	64.0	0.96	0.05
		BMI (kg/m^2^)	21.8 (1.8)	21.9	21.7 (2.4)	21.6	0.81*	0.16*
	Overweight/obese^2^ (n=24)	Height (cm)	176.1 (10.6)	177.5	175.1 (13.4)	179.5	0.96	0.15
		Weight (kg)	84.5 (13.9)	83.5	81.1 (14.6)	80.0	0.93	**<0.01**
		BMI (kg/m^2^)	27.1 (2.1)	26.2	26.3 (2.3)	25.9	0.40*	0.14*

* Non-parametric equivalent test used (spearman correlation and Wilcoxon signed ranks test).

^1^ BMI<25 kg/m^2^, or BMI less than age-specific cut-offs if under 18 years.

^2^ BMI ≥25 kg/m^2^, or BMI greater than age-specific cut-offs if under 18 years.

Significant p-values (p<0.05) shown in boldface.

SD - standard deviation

Correlations between self-reported and measured values for height and weight were high (Table 2); correlation was equal to or greater than 0.92 for all values and sub-categories with the exception of height among females, with correlation of 0.84. Correlation of self-reported and measured BMI was moderately high for all sub-groups with the exception among overweight or obese individuals, in which correlation was only 0.40.

Overall, 52% of participants accurately self-reported their height (within 2cm), 30% under-reported, and 18% over-reported their height (Table 3). There was a tendency for more males than females to over-report their height (p=0.16).

**Table 3:** Proportion of participants who accurately, under- and over-reported their height and weight stratified by sex, age group, and obesity classification

			**Accurate**^**1**^		**Under-report**^**2**^		**Over-report**^**3**^		**p-value**^**4**^
			**n**	**%**	**n**	**%**	**n**	**%**	
Height		Overall	35	52	20	30	12	18	
	Sex	Males	17	50	8	24	9	26	0.16
		Females	18	55	12	36	3	9	
	Age group (years)	16-19	16	48	10	30	7	21	0.72
		20-29	19	56	10	29	5	15	
	Obesity classification	Non-overweight^5^	22	54	11	27	8	20	0.79
		Overweight/obese^6^	13	50	9	35	4	15	
Weight		Overall	23	34	35	52	9	13	
	Sex	Males	15	44	12	35	7	21	**0.01**
		Females	8	24	23	70	2	6	
	Age group (years)	16-19	13	39	16	48	4	12	0.74
		20-29	10	29	19	56	5	15	
	Obesity classification	Non-overweight^5^	17	41	18	44	6	15	0.24
		Overweight/obese^6^	6	23	17	65	3	12	

^1^*Accurate* defined as self-report less than two units (kg or cm) different from measured value.

^2^*Under-report* defined as self-reported height or weight two or more units (kg or cm) less than measured value.

^3^*Over-report* defined as self-reported height or weight two or more units (kg or cm) more than measured value.

^4^ Fisher’s exact test. Significant p-values shown in bold.

^5^ BMI<25 kg/m^2^ or less than age-specific cut-offs if under 18 years.

^6^ BMI ≥25 kg/m^2^ or more than age-specific cut-offs if under 18 years.

Significant p-values (p<0.05) shown in boldface.

Overall, 34% of participants accurately self-reported their weight (within 2 kg), 52% under-reported and 13% over-reported their weight (Table 3). Significantly more females (70%) than males (35%) under-reported their weight by at least two kilograms (p=0.01). Compared to non-overweight participants, overweight/obese participants were less likely to accurately report weight and more likely to under-report weight, but this was non-significant (p=0.24).

In total, 3 (9%) males and 9 (27%) females inaccurately self-reported their height by five centimetres or more (p=0.05), and 12 (35%) males and 5 (15%) females inaccurately self-reported their weight by five kilograms or more (p=0.06). Overweight/obese participants were more likely to misreport their weight by at least 5kg than non-overweight participants (42% vs 15%, p=0.01).

### Classification of overweight/obesity

Based on self-report data, 22 (33%) participants were classified as overweight or obese, compared to 26 (39%) participants based on measured data (p=0.29). In total, 59 (88%) were correctly classified. The sensitivity of self-reported data was 77% (95%CI 56%-91%) and specificity was 95% (95%CI 83%-99%).

## Discussion

In this study of young people attending a music festival, we determined the feasibility and accuracy of collecting self-reported height and weight. Our results confirm that at a group level, self-report measures in a community-based setting is a useful tool for estimating the prevalence of overweight and obesity, particularly when impractical to take independent measurements.

Three-quarters of survey participants provided both self-report height and weight. In the remainder, the majority reported not knowing their height and/or weight, with only a few missing values, although the “don’t know” category might have also included some refusals. Of concern, participants who did not self-report their height or weight were systematically different from those who did; they were more likely to be female, younger, and less educated. Because height and weight measurements were not taken for all participants, we could not determine whether self-reporting height and weight was influenced by bodyweight status. Further research is needed to explore if there are biases in bodyweight status influencing willingness and ability to self-report height and weight in a community-based setting.

Self-reported and measured height did not significantly differ, and approximately half of males and females reported their height within two centimetres of measured height. However, females were more likely than males to underreport their height, and approximately one quarter of females misreported their height by more than five centimetres, compared to only nine percent of males. Previous studies have observed over-report of height [6,7] or decreased accuracy of height with increasing age, perhaps due to decreasing opportunities to regularly measure height or changes in height over time [4,27]. Of note, we did not detect a difference in accurate report by age group in our study.

The difference between self-reported and measured weight was more pronounced than for height, particularly among females and overweight or obese individuals. Although one third of all individuals reported their weight within two kilograms of measured weight, a notable 35% of males and 15% of females misreported their weight by five or more kilograms. Females were more likely than males to under-report their weight. Our results are consistent with previous studies reporting systematic under-reporting of weight by females and overweight/obese individuals [4,12]. It has been postulated that social desirability bias may explain the underreporting of weight, particularly among females and overweight/obese individuals [8,28]. However, other research that has included a measure of social desirability has challenged this notion [26,28].

In this study we found that self-report height and weight is a reasonable predictor of overweight and obesity among young people, particularly when pertaining to population-level applications such as monitoring trends in overweight, program evaluation, and advocacy for funding [12]. Notwithstanding inaccuracies in the self-reporting of weight, the effect on BMI was small, with the median difference less than one unit. Sensitivity of classification of overweight/obesity was around 77% − similar to the 70% found in 15–19 year olds participants of the Australian National Health Survey [6]. Nonetheless, in this sample, approximately two-fifths of overweight/obese individuals would have been incorrectly classified as non-overweight based on self-report (false negatives), which is consistent with previous findings [12]. Methods to limit this inaccuracy and bias might include a correction algorithm to account for generalised misreporting based on certain characteristics [2,11,29], periodically measuring a sub-sample, or where feasible, advising participants ahead of time to weigh and measure themselves before participating [30].

Obesity prevention and control is a national priority in Australia, with increasing millions of dollars being invested to its cause [31]. Community-based settings, including web-based studies, are ideal alternatives to traditional means of population-based recruitment in order to both inform and evaluate obesity interventions – particularly among young adults who are difficult to access through household telephone surveys and school settings. They provide novel means to reach a large number of people, including hard-to-reach populations [32]. In these settings self-reported anthropometric measures are the most practical means to define and estimate the prevalence of overweight and obesity. To our knowledge, this is the first study to investigate accuracy of self-report in a community-based sample; our findings confirm that it is possible to use self-report data to estimate and monitor trends in prevalence of overweight and obesity, particularly when bias is expected to remain constant.

This study has a number of limitations. First, the results are based on a convenience-sample and may not be representative of young people in Australia. Second, the sample size for the independent measurement of height and weight was relatively small and may have limited our ability to detect differences and associations with self-reported height and weight. This sample size was limited by the use of only one measuring station, and in the future we recommend more stations are utilised to obtain a larger sample. Third, small differences in self-reported and measured values may be attributable to non-differential measurement bias; multiple researchers were responsible for taking measurements throughout the day and the recruitment day was characterised by high temperatures, reaching up to 40 degrees Celsius. Some participants may have been dehydrated while others were drinking large quantities of water (the study team was distributing water); both factors may have impacted on the accurate measurement of weight. Natural daily weight fluctuation may also partly explain some weight discrepancies; at the festival measurements were taken between 10am-3pm, which may differ to participants’ customary time for weighing themselves. Fourth, selection bias may have been introduced because individuals were not systematically randomised to have their height and weight measured by a researcher; there was potential unrecognised selection bias by the investigators inviting participants to be measured, as well in participants who declined to be measured. However, no demographic differences were identified between those with and without measurements taken. Further research is needed to confirm findings in a larger sample and to see whether a convenience music festival audience differs from the general young adult population in terms of self-reported weight and height accuracy.

## Conclusions

In conclusion, our results suggest that the majority of young people participating in a risk behavioural survey are able and willing to self-report their height and weight. The classification of overweight/obesity based on derived self-report BMI has high sensitivity against independent measurement in this setting. Given the wide reach and efficiency of surveys including self-reported measures, these results suggest that that self-reported height and weight are a suitable proxy to estimate the prevalence of overweight and obesity by BMI in a community-based sample of young people, but some underestimation of overweight/obesity is likely and should be taken into consideration.

## Competing interest

The authors declare that they have no competing interests.

## Authors’ contributions

The following co-authors have contributed to the work: AB in data collection, data analysis, manuscript preparation and manuscript review; AP in study design, manuscript preparation and manuscript review; MG in data analysis and manuscript review; RFP in manuscript preparation and manuscript review; MSCL in study design and manuscript review; MH in study design, manuscript preparation and manuscript review. All authors read and approved the final manuscript.

## Pre-publication history

The pre-publication history for this paper can be accessed here:

http://www.biomedcentral.com/1471-2288/12/175/prepub
